# Evolutionary genetics of personality in the Trinidadian guppy I: maternal and additive genetic effects across ontogeny

**DOI:** 10.1038/s41437-018-0082-1

**Published:** 2018-05-17

**Authors:** Stephen John White, Alastair James Wilson

**Affiliations:** Centre for Ecology and Conservation, University of Exeter (Penryn Campus), Cornwall, TR10 9FE UK

**Keywords:** Evolutionary genetics, Behavioural ecology

## Abstract

Among-individual variation in behaviour is a widespread phenomenon, with several frameworks developed to explain its existence. Maternal effects, which can have significant influence over evolutionary processes, are an understudied source of behavioural variation. Maternal effects are not necessarily static, however, since their importance can change over offspring ontogeny, typically declining with age relative to additive genetic effects. Here, using a quantitative genetics approach, we test the prediction that maternal effects will influence age-specific risk-taking behaviour in Trinidadian guppies, *Poecilia reticulata*. Individuals were subject to a single open-field trial as juveniles and up to four repeat trials as adults, with five traits indicative of risk-taking behaviour measured in each trial. We then partitioned phenotypic variance into additive genetic (*V*_A_) and maternal identity (*V*_M_) components, in addition to testing brood size and maternal weight as specific sources of maternal effects. We found that *V*_M_ had significant influence over juvenile traits, with very low *V*_A_ estimates. Whereas, in adults, all traits were significantly heritable, with little support for *V*_M_. We also found a strong influence of maternal traits on juvenile behaviours as predicted, with significant, albeit smaller, effects found in adults. Maternal weight was heritable and itself subject to maternal effects. Thus, maternal weight is a likely source of maternal genetic effects that are expected to alter response to selection on personality in this system. More generally, our study highlights that while maternal effects can be an important source of personality variation, this varies over ontogeny of offspring.

## Introduction

Among-individual variation in behaviour, or personality, has been well documented in a large number of animal species. No longer considered as simply noise around the mean, there have been multiple adaptive frameworks developed to try to explain the maintenance of personality variation. These frameworks include frequency dependent selection (Wolf et al. 2008), fluctuating selection (Dingemanse et al. 2004; Le Coeur et al. 2015), pace-of-life syndrome (Biro and Stamps 2008; Réale et al. 2010) and state-dependent feedback loops (Luttbeg and Sih 2010; Sih et al. 2015). Although there is some empirical support for each of these, it is not clear that a single explanation will apply to all cases. Furthermore, these adaptive explanations for personality variation implicitly assume a genetic basis to the variation. This is because any selection response depends on heritable variation, while simple linear and/or stabilising forms of selection are not expected to erode personality variance if it is completely environmentally induced. While evidence for additive genetic variation underpinning repeatable behavioural traits is now growing, few studies have considered the potential role of maternal effects in driving among-individual differences. If mothers differ in specific traits that influence offspring behaviour (e.g., aspects of maternal care), this could also generate among-individual variation in offspring traits. Here, we seek to address this gap, by evaluating maternal effects as both a potential cause of bias and a further source of evolutionarily significant variation in a study of age-specific personality in the Trinidadian guppy, *Poecilia reticulata*.

Personality traits such as boldness and aggression have been linked to survival and reproductive success (Smith and Blumstein 2008; Ariyomo and Watt 2012). Given this association with fitness-related traits, if personality traits exhibit sufficient additive genetic variation, then they have the potential for evolution. However, we might predict that—at least where selection is linear and/or stabilising—genetic variance for personality should diminish over time (Falconer and Mackay 1996; Kruuk et al. 2008). Despite this expectation of reduced variation due to selection, genetic variation in personality traits has been quantified in a range of taxa including fish (Dingemanse et al. 2012; Ariyomo et al. 2013), birds (Drent et al. 2003; Brommer and Kluen 2012) and mammals (Brent et al. 2014; Johnson et al. 2015; Petelle et al. 2015). A recent review of published studies concluded that the average heritability of personality traits was as high as 0.52 (Dochtermann et al. 2015). This estimate is perhaps potentially misleading as additive genetic variance estimates were scaled by among-individual phenotypic variance only (which logically follows the definition of personality variation as being among-individuals, but means within-individual behavioural variation from plasticity and/or measurement error is excluded). Nonetheless, evidence of genetic variance underpinning personality traits is certainly growing, and it is in this context that explanations have been sought for the maintenance of consistent among-individual differences in behaviour.

While quantitative genetic studies have largely sought to test the additive genetic basis of variation, additional factors are known to influence development and/or expression of personality, including aspects of the social environment (Moretz et al. 2007; Piyapong et al. 2010; King et al. 2015), abiotic variables such as temperature (Biro et al. 2010; Briffa et al. 2013) and availability of food or other resources (Dingemanse et al. 2004; Le Coeur et al. 2015). Here we consider maternal effects as a potential source of variation in behaviour. Maternal effects occur when the maternal phenotype influences the offspring phenotype, above and beyond the normal inheritance of genes (Mousseau and Fox 2008). This can occur through a range of pathways, such as provisioning of food and other types of parental care (Reznick et al. 1996; Hunt and Simmons 2002; D’Amore et al. 2015), or exposure to maternal hormones during development (Tobler and Sandell 2007; Groothuis et al. 2008; Rokka et al. 2014; Hinde et al. 2015). Although some maternal effects on offspring behaviour are known (Duckworth et al. 2015; Kasper et al. 2017; Storm and Lima 2010; Taylor et al. 2012), most studies have focussed on physiology (Bacigalupe et al. 2007; Tobler et al. 2007), life history (Hunt and Simmons 2002; Bashey 2006) and growth (Wilson et al. 2005).

Despite maternal effects having thus far remained an understudied source of among-individual variation in behaviour, they can be important for our understanding of the evolution of personality traits for two major reasons. First, failing to consider maternal effects can result in upwardly biased estimates of heritability (*h*^2^) and so to over-prediction of responses to selection (Falconer and Mackay 1996; Kruuk 2004; Wilson et al. 2010). Secondly, maternal effects can themselves have a significant genetic (among-mother) basis of variation, with important consequences for the evolutionary dynamics of offspring traits. For instance, maternal genetic effects can cause time-lagged responses to selection, even if the offspring trait itself has little or no additive genetic basis (Räsänen and Kruuk 2007). Furthermore, correlations between maternal genetic and additive genetic effects can either constrain or facilitate the response of offspring traits to selection (Kirkpatrick and Lande 1989; Räsänen and Kruuk 2007; Charmantier et al. 2013). Although maternal genetic effects on personality have received little attention to date, their presence is actually implicit in ideas such as ‘adaptive priming’, in which maternal effects are viewed as having evolved to increase offspring fitness by priming their behaviour for an anticipated local environment (Reddon 2011; Mainwaring and Hartley 2013; Rokka et al. 2014).

Maternal effects can thus be a source of offspring behavioural variation and can act to alter their evolutionary trajectories, yet the strength of these effects can change over the ontogeny of offspring (Arriero et al. 2013; Andree et al. 2015; Houde et al. 2015; Van Leeuwen et al. 2015). Previous studies have shown that as individuals grow and mature, the relative importance of environmental and additive genetic variance components often tends to increase at the expense of maternal effects (Wilson and Réale 2005; Lindholm et al. 2006; Dibattista et al. 2009). In light of this, a more complete picture of how maternal effects influence personality traits requires such effects to be measured at multiple points in the offspring’s life. It would also be valuable to determine the mechanisms and maternal traits through which any maternal effects are mediated. Although the possibilities are numerous in this regard, the commonly reported link between size and boldness traits in fish (Brown and Braithwaite 2004), coupled with well-documented maternal effects on size (Bashey 2006; Leblanc et al. 2014; Murphy et al. 2014; Einum and Fleming 1999) suggests one very plausible mechanism. If maternal effects on offspring behaviour are present and mediated by impacts on offspring size or growth, then we expect (a) significant effects of standard length (SL) on behaviour and (b) reduced support for maternal trait effects with its inclusion in the analysis.

Here, we test the importance of maternal and additive genetic effects on risk-taking behaviours expressed during an open-field trial (OFT) and whether this changes over ontogeny in *P. reticulata*. This species provides an ideal model as it is easily bred in captivity (facilitating a quantitative genetic approach), while differential yolk provisioning of eggs is a known source of maternal effects on offspring size/growth (Reznick et al. 1996; Bashey 2006). Here, we ask whether maternal effects contribute to among-individual variation in juvenile risk-taking behaviour. If so, we go on to ask how such effects change as offspring reach maturity. In addition, we test whether these maternal effects on offspring personality are mediated by offspring size. In doing so, we build on the results of our previous study that demonstrated that risk-taking behaviours, putatively indicative of shy-bold type personality variation and behavioural stress ‘coping style’, are repeatable in this population and can be classed as personality traits (White et al. 2016).

Using an animal model framework, we test for maternal effects arising specifically from maternal weight (at offspring birth) and brood size. These traits are expected to provide insight into among-female variation in resource allocation. We also estimate non-specific maternal effects (i.e., arising from unknown aspects of maternal phenotype) and additive genetic effects using a standard variance partitioning approach. We predict, firstly, that maternal effects on risk-taking behaviour will be present (such that failure to model them will lead to inflated *h*^2^ estimates). Secondly, that the relative importance of maternal and additive genetic effects will change across ontogeny, with the former being less important for determining adult offspring personality. And thirdly, these maternal effects will be mediated, in part, through direct impacts on offspring size that in turn have consequences for behaviour. Finally, we test for genetic variance in two suspected sources of maternal effects, female weight and brood size. If these traits are both heritable and a source of maternal effects, it follows that they are a source of maternal genetic effects expected to have important consequences for the evolutionary dynamics of personality.

## Materials and methods

### Fish husbandry and breeding

Fish used were from a captive population of *P. reticulata* maintained at the University of Exeter, Penryn campus fish facility. The population is descended from wild fish caught in 2008 from the lower Aripo River, Trinidad (ca. 18–24 generations ago) and has been maintained at an effective population size of several thousand, with no deliberate selection or inbreeding. Data were obtained for 653 juvenile and 831 adult guppies, spread across a three generation pedigree (parental, G1 and G2) using a paternal half-sib breeding design. See supplemental appendix 1 for details of the breeding methodology and associated husbandry and supplemental appendix 2 for a visualisation of the pedigree structure.

Juvenile fish were initially kept in full-sib family groups, with each family housed in a 2.8 L tank. These fish were untagged, so identification of individuals was not possible. All juvenile family groups were kept on a single water supply to prevent tank effects arising from water chemistry differences. One week after the juvenile OFT, all juveniles were moved to 15 L ‘grow on tanks’, still in family groups. Note that family sizes were not reduced to a common standard, such that maternal brood size directly determines early life density. To the extent that early rearing density influences individual behaviours, our estimation of maternal brood size effects (see below) will therefore integrate across pre-natal and post-natal effects. In other words, under our experimental conditions, a significant effect of brood size could occur if early rearing density influences offspring behaviour but pre-natal brood size does not.

At an average age of 132 days (range 59–226), the now mature fish were tagged with visible implant elastomer (under anaesthetic, using a buffered solution of MS222) for individual identification, and transferred to mixed family groups of size 16—8 males and 8 females. Variation in age is controlled for in all models of behaviour (see statistical methods below) and arose because groups were necessarily established sequentially as sufficient fish from multiple families reached a size at which tagging was deemed a safe procedure for the animals. Thus, each adult group comprised a mix of mature fish available from all broods in which individuals are sufficiently large enough to tag. By mixing fish among families in this way, we reduce the potential for common environment effects to upwardly bias the maternal and/or genetic parameters estimated.

### Phenotyping of fish

At an average age of 49.8 days (range 35–55), each untagged individual from each brood was subject to a single OFT (described further below) in what constitutes the juvenile measure. One week after tagging, all G1 adult fish experienced four repeat OFTs over a 2-week period (with at least 48 h between trials). For G2 fish, four behavioural trials were also conducted over a 2-week period, but we performed only two OFTs per individual. These were alternated with two ‘emergence trials’ similar to those described in White et al. (2016), the data from which are not included in the present study. G1 fish therefore had one juvenile OFT measure and four adult OFT measures. G2 individuals had one juvenile measure and two adult measures.

OFT data were also collected on the parental generation of fish prior to beginning of the breeding program (again, four repeats separated by a minimum of 48 h over a 2-week period). Note that the age of the parental generation fish was unknown (but all were mature adults as inferred from external morphology). The temperature of the OFT tank water was measured at the end of each behavioural trial allowing subsequent statistical control for variation around the mean of 23.7 °C. Additionally, SL (measured from snout to caudal peduncle, mm) and weight of each fish was recorded after each trial before fish were returned to their group housing.

### Open-field trials

We followed the OFT methodology described by White et al. (2016). Briefly, an individual fish was introduced to an empty arena (30 cm × 20 cm × 20 cm tank filled to a depth of 5 cm and lit from below). Using a digital camera and Viewer software (www.biobserve.com), fish movement was then tracked over a 4 min 30 s period (after 30 s acclimation period). From the tracking data, we extracted the *tracklength* as the total distance swum (cm) by the focal fish, the percentage of time spent active, which we defined as moving at >4 cm s^−1^ (*activity*), the percentage of the tank floor area that was explored during the trial (henceforth *area covered*), the number of times each individual ‘froze’, defined in practice as the velocity dropped below 4 cm s^−1^ for >2.5 s (henceforth *freezings*) and the amount of time spent in the inner, putatively ‘risky’, zone of the tank (henceforth *time in middle zone*). For the last of these, the floor area of the tank was partitioned into middle and outer zones of equal size using the Viewer software. Water in the OFT tank was replaced between each group, and any effect of chemical cue build-up is controlled statistically (see statistical methods).

Note, the OFT is a standard approach for quantifying among-individual behavioural variation (or personality), in small fishes (Oswald et al. 2013; Boulton et al. 2014), including guppies (Burns 2008; Diaz Pauli et al. 2015). The traits measured in the present study have been found to all effectively assay a shy/bold type axis of behavioural variation in the sheepshead swordtail *Xiphophorus birchmanni*, a species closely related to the guppy (Boulton et al. 2014). Broadly similar patterns were found in a previous study of this population, with all traits being repeatable (a prerequisite for heritability) with putatively bolder (or risk-prone) fish tending to explore more area and spend more time in the inner zone (White et al 2016). However, *tracklength* and *activity* also appear to capture variation in behavioural stress response (or ‘coping style’) that does not quite conform to predictions made under a simple shy-bold continuum (White et al 2016). So, while simulating predation events in the lab has shown that all traits respond plastically to increases in perceived predation risk (Houslay et al. 2018), under a simple shy-bold paradigm we would predict, for instance, a strong positive correlation among-individuals between *tracklength* and *area covered* that is not present in our previous behavioural studies (White et al 2016; Houslay et al 2018). In the present study, we present univariate analyses of five observed traits that we refer to collectively as risk-taking behaviours. We note that while the OFT traits analysed here should not be viewed as independent of each other, but nor are they completely equivalent, and thus redundant, proxies of a single axis of personality variation. Full investigation of the covariance structure among these behaviours is presented in our companion paper based on the same data (White and Wilson, in press), and we refer the interested reader to that for more detail.

### Statistical methods

Univariate mixed models for each of the five OFT traits were fitted to both juvenile and adult data sets using a restricted maximum likelihood framework in ASReml-R (Butler et al. 2009). *freezings* and *time in middle zone* in both juvenile and adult data were square root-transformed to better meet assumptions of homoscedasticity and normality of residuals (which were checked, and found to be reasonable, by visual inspection of model residuals). All traits were then mean-centred and rescaled to SD units prior to analysis to allow direct comparison of variance components for each trait.

In both juvenile and adult models, *temperature*, *age*, *order caught* and *generation* were fitted as fixed effects to control for sources of variance not relevant to our hypotheses. *Temperature* and *age* were modelled as continuous linear effects*. Order caught* is the order in which fish were caught from their home tank prior to the OFT. Although we acknowledge that *Order caught* could itself vary consistently among individuals as consequence of either fish behaviour in the home tank or unconscious selection by the researcher, we elected to include it here to control for among-individual variation in disturbance and any build-up of chemical cues in the OFT tank over the course of measuring a brood/group. Slight differences between the breeding protocol and housing between the parental, G1 and G2 generations (see supplemental appendix 1) are controlled for with the *generation* fixed effect.

The adult models had an additional fixed effect of *repeat*, to control for potential habituation to the OFT procedure over the repeat measures. Note that while sexual dimorphism in behaviour is present (White and Wilson, in press), sex was known in adults only, so in order to allow direct comparison between juvenile and adult results, we present results from models that do not include a fixed effect of sex at the adult life stage. This is appropriate to the hypotheses being tested, with model parameter estimates thus being interpretable as averaged across sexes in both juveniles and adults (but see White and Wilson, in press).

Conditional F statistics were used for ascertaining significance of fixed effects. For variance components, we assumed a *χ*^2^ statistic to be equivalent to twice the difference in log-likelihood between full and reduced models with degrees of freedom equivalent of the number of parameters being tested. A 50:50 mix of *χ*^2^_0_ and *χ*^2^_1_ (henceforth *χ*^2^_0,1_) is also assumed when testing a single variance component, as recommended by Visscher (2006).

### Estimating additive genetic and maternal effects over ontogeny

For each age-specific trait, we partitioned the phenotypic variance (*V*_P,_ conditional on fixed effects) into components attributable to maternal effects, additive genetics and other environmental sources of variation. Maternal effects were estimated using the ‘hybrid’ strategy suggested by McAdam et al. (2013) in which we: (i) fitted the maternal traits of *brood size* and *maternal weight* at offspring birth (and their interaction) as fixed effects to test the hypothesis that these maternal traits affect personality (in addition to known effects on growth and life history; Shikano and Taniguchi 2005; Bashey 2006); and, (ii) included a random effect of *maternal identity* to capture variance in maternal ‘performance’ for offspring behaviour (*V*_M_). Both *maternal weight* and *brood size* were mean-centred and transformed into SD units (*maternal weight*, mean = 0.45 g, SD = 0.13; *brood size* mean = 17.21, SD = 6.65). Additive genetic variance (*V*_A_) was estimated by including a random effect of individual identity linked to the pedigree following a standard maternal effect animal model formulation (Wilson et al. 2009). For adult traits, two additional random effects were included: a permanent environment effect (with variance *V*_PE_) to account for repeat measures on individuals, and a housing group effect (with variance *V*_GROUP_) representing the social and physical environment experienced by each individual. Additional random effects in the adult models do not mean that additional phenotypic variance is modelled relative to the juveniles, but rather that additional partitions of *V*_P_ are made. Thus for juveniles, all environmental variance is partitioned as residual variance (*V*_R_). Conversely, in adults, *V*_R_ represents within-individual variance from plasticity and/or measurement error with non-genetic among-individual variance separately partitioned as *V*_PE_. Thus, while the magnitudes of additive and maternal genetic variances can be compared across age classes, comparison of residual variance would not be biologically meaningful and estimation of trait repeatabilities is not possible in juveniles.

Narrow sense heritabilities (*h*^2^ = *V*_A_/*V*_p_) were calculated for juveniles and adults, and maternal identity effects were similarly standardised to a proportion of total phenotypic variance (*m*^2^ = *V*_M_/*V*_P_). In all cases, phenotypic variance was defined conditional on fixed effects and calculated as the sum of the estimated variance components. For each trait, we estimated *h*^2^ and *m*^2^ under the ‘full’ model (including fixed effects as described below), but also compared the fit of this model to a ‘null’ that included neither additive nor maternal identity effects, and two intermediate models containing either additive or maternal identity effects only. We used likelihood ratio tests to make comparison among these models where possible. However, since the two intermediate models are not nested, then to discriminate among the set of four models considered for each age-specific trait, we also computed and compared AIC (Akaike information criterion).

### Does offspring length mediate maternal effects on offspring behaviour?

In order to test whether maternal effects influence offspring risk-taking behaviour through offspring size, we refitted the above full models for juveniles and adults with an additional fixed effect of offspring SL.

### Estimating maternal genetic effects

Finally, given our hypothesis that maternal effects on offspring behaviour could arise through causal dependence on maternal weight and/or brood size, we tested these traits for both (among-female) heritable variation and maternal effects. The former is of interest since, if these traits do causally influence offspring behaviour, then heritable variation in them will be a source of maternal genetic effects. The latter is potentially important because cascading maternal effects (*sensu* McGlothlin and Galloway 2013) arise if maternal effects on offspring are mediated by traits that themselves have a maternal influence (i.e., there is a grand-maternal influence on the offspring). We fitted an animal model of *female weight* using all available measures of adult females and a fixed effect of age (as a cubic function to allow for non-linear growth) in addition to the mean. Random effects as described above were used to partition variance into *V*_A_, *V*_M,_ *V*_PE_ and *V*_R_. The *Brood size* model was similar but we included *female weight* as a fixed covariate, enabling us to condition our estimates on the known increase in fecundity with female size (Reznick 1983). This model therefore tests for genetic variance in *Brood size* after accounting for female body size.

## Results

### Additive genetic and maternal effects on offspring behaviour over ontogeny

Model comparisons provided strong evidence for among-family variance consistent with additive genetic and/or maternal identity effects across all traits in juveniles and adults. Comparison of model likelihoods (shown in Table 1) indicates that the full (*V*_A_ + *V*_M_) model is a significantly better fit than the null model in every case (*χ*^2^_2_ ranges from 13.6 to 69.9, all *P* = <0.001; Supplemental Table 1). In juveniles, support for maternal identity effects comes from the fact that the full (*V*_A_ + *V*_M_) model is significantly better than the *V*_A_-only model for *tracklength*, *activity*, *area covered* and *freezings* (*tracklength* *χ*^2^_0,1_ = 8.17, *P* = 0.002, *activity* *χ*^2^_0,1_ = 7.78, *P* = 0.003, *area covered* *χ*^2^_0,1_ = 4.04, *P* = 0.022, *freezings* *χ*^2^_0,1_ = 4.31, *P* = 0.019). For *time in middle zone*, this comparison is marginally non-significant (*χ*^2^_0,1_ = 2.62, *P* = 0.053). Conversely, the full model was not significantly better than the *V*_M_-only model for any trait, and all estimates of *V*_A_ in the full model are bound to zero. In accordance with these results, the *V*_M_-only model is preferred (i.e., lowest AIC) for all juvenile behaviours. Thus, we conclude maternal effects are the main driver of among-family variation in juvenile traits.

**Table 1:** Comparison of null, *V*_A_ only, *V*_M_-only and full (*V*_A_ + *V*_M_) models for all risk-taking traits in juveniles and adults

Trait	Juvenile				Adult			
	Model	AIC	ΔAIC	Loglik	Model	AIC	ΔAIC	Loglik
Tracklength	Null	357.99	45.40	−178.00	Null	1485.6	36.4	−739.8
	*V*_A_	320.77	8.17	−158.38	*V*_A_	1454.4	5.2	−723.2
	*V*_M_	312.60	0.00	−154.30	*V*_M_	1449.2	0	−720.6
	*V*_A_ + *V*_M_	314.60	2.00	−154.30	*V*_A_ + *V*_M_	1449.4	0.2	−719.7
Activity	Null	380.73	52.44	−189.37	Null	1885.7	39	−939.8
	*V*_A_	336.07	7.78	−166.04	*V*_A_	1846.7	0	−919.4
	*V*_M_	328.29	0.00	−162.15	*V*_M_	1859.8	13.1	−925.9
	*V*_A_ + *V*_M_	330.29	2.00	−162.15	*V*_A_ + *V*_M_	1847.6	0.9	−918.8
Area covered	Null	691.96	67.90	−344.98	Null	2096.3	19.4	−1045.1
	*V*_A_	628.10	4.04	−312.05	*V*_A_	2076.9	0	−1034.4
	*V*_M_	624.06	0.00	−310.03	*V*_M_	2095.4	18.5	−1043.7
	*V*_A_ + *V*_M_	626.06	2.00	−310.03	*V*_A_ + *V*_M_	2078.9	2.0	−1034.4
Time in middle	Null	720.80	14.57	−359.40	Null	2048.5	11.6	−1021.2
	*V*_A_	707.44	1.21	−351.72	*V*_A_	2036.9	0	−1014.5
	*V*_M_	706.23	0.00	−351.12	*V*_M_	2050.2	13.3	−1021.1
	*V*_A_ + *V*_M_	708.23	2.00	−351.12	*V*_A_ + *V*_M_	2038.9	2.0	−1014.5
Freezings	Null	529.82	33.95	−263.91	Null	2317.9	25.1	−1155.9
	*V*_A_	500.19	4.31	−248.10	*V*_A_	2292.8	0	−1142.4
	*V*_M_	495.88	0.00	−245.94	*V*_M_	2314.5	21.7	−1153.3
	*V*_A_ + *V*_M_	497.88	2.00	−245.94	*V*_A_ + *V*_M_	2294.8	2.0	−1142.4

Shading denotes the preferred model in each case as determined by minimum AIC score. ΔAIC is the difference in AIC between every model with the preferred model. Fixed effects of temperature, age, order caught and generation were included in both juvenile and adult models with an additional fixed effect of repeat in adult models

For adult traits, the *V*_A_-only model is the preferred model for all but one trait. For *tracklength*, the *V*_M_-only model is preferred to the *V*_A_-only model (ΔAIC = 5.2) but is only marginally better than the full model (ΔAIC = 0.2). We thus conclude maternal identity effects are important for *tracklength* in adults. For *area covered*, *time in middle zone* and *freezings*, the estimate of *V*_M_ is bound to zero in the full model (resulting in no improvement of log-likelihood). This suggests that the among-family variance is largely driven by additive genetic effects, the preference for the *V*_A_-only model being reflected by ΔAIC ≥2 for all other models (Table 1).

Given the expectation that dropping either *V*_A_ or *V*_M_ could lead to upward bias of the retained component, we elected to estimate *h*^2^ and *m*^2^ from the full model for all traits (while acknowledging this necessarily means greater uncertainty on all parameter estimates; Table 2). Indeed, omitting *V*_M_ leads to higher (and statistically significant) heritability estimates for juvenile traits (range from 0.173 to 0.615; see Supplemental Table 2) when compared to the full model (zero for all juvenile behaviours; Table 2). In adults, *V*_M_ was bound to zero in three of the five traits in the full model (Table 2) and there is a pattern of *m*^2^ being higher in juveniles (range 0.081–0.254, median = 0.170) than in adults (range 0.00–0.10, median = 0.00). Where *V*_M_ = 0, dropping the maternal identity has no impact on estimated heritability. In adult *tracklength* and *activity*, heritability is increased by dropping the maternal identity effects (as in the juvenile traits, though to a much lesser extent; supplemental Table 2).

**Table 2:** Estimated variance components and their corresponding ratios to phenotypic variance (conditional on fixed effects)

Trait	*V*_A_	*V*_M_	*V*_PE_	*V*_Group_	*V*_R_	*h* ^2^	*m* ^2^	pe^2^	Group^2^
**Juvenile**									
*Tracklength*	0.000 (−)	0.096 (0.033)	—	—	0.469 (0.028)	0.000 (−)	0.170 (0.049)	—	—
*Activity*	0.000 (−)	0.134 (0.043)	—	—	0.474 (0.028)	0.000 (−)	0.220 (0.057)	—	—
*Area covered*	0.000 (−)	0.257 (0.077)	—	—	0.756 (0.045)	0.000 (−)	0.254 (0.059)	—	—
*Time in middle*	0.000 (−)	0.080 (0.037)	—	—	0.910 (0.053)	0.000 (−)	0.097 (0.039)	—	—
*Freezings*	0.000 (−)	0.113 (0.040)	—	—	0.634 (0.037)	0.000 (−)	0.151 (0.047)	—	—
**Adult**									
*Tracklength*	0.056 (0.045)	0.079 (0.037)	0.215 (0.034)	0.043 (0.019)	0.423 (0.014)	0.068 (0.055)	0.097 (0.042)	0.263 (0.042)	0.053 (0.023)
*Activity*	0.164 (0.055)	0.021 (0.023)	0.182 (0.040)	0.023 (0.014)	0.504 (0.017)	0.184 (0.058)	0.023 (0.026)	0.204 (0.046)	0.026 (0.015)
*Area covered*	0.167 (0.050)	0.000 (−)	0.114 (0.037)	0.155 (0.045)	0.587 (0.020)	0.163 (0.046)	0.000 (−)	0.111 (0.038)	0.151 (0.038)
*Time in middle*	0.158 (0.056)	0.000 (−)	0.237 (0.044)	0.026 (0.015)	0.534 (0.018)	0.165 (0.055)	0.000 (−)	0.248 (0.048)	0.027 (0.016)
*Freezings*	0.202 (0.054)	0.000 (−)	0.093 (0.039)	0.021 (0.013)	0.662 (0.022)	0.206 (0.051)	0.000 (−)	0.096 (0.041)	0.022 (0.013)

Estimates were made under the full model for each juvenile and adult behaviour and SEs are shown in parentheses (but note where parameters were bound to zero, no SE is estimatable). Fixed effects of temperature, age, order caught and generation in both juvenile and adult models and an additional fixed effect of repeat in adult models

Although not directly relevant to our primary hypothesis, we also note that post hoc testing of adult traits indicated that among-group variance was significant for all adult traits (potentially indicative of social effects on behaviour). Additionally, permanent environment effects accounted for 10–26% of phenotypic variance in adult traits (Table 2), highlighting the importance of additional (but currently unknown) sources of among-individual behavioural differences.

**Fig. 1:**
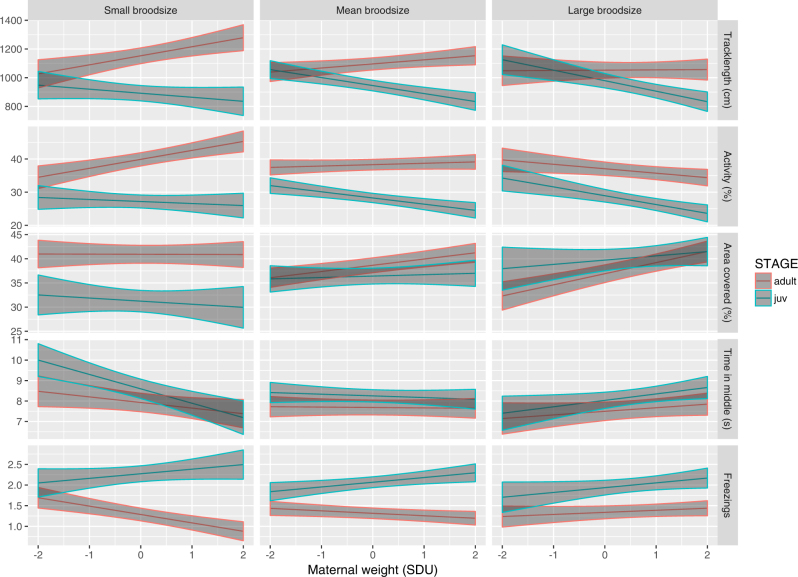
Predicted relationships between *Maternal weight* and offspring behaviour for each OFT. Predictions are shown for juvenile (blue) and adult (red) offspring from small (*n* = 5), mean (*n* = 17.21) and large (*n* = 25) brood sizes. Shaded areas indicate ± 1 SE around the predicted behavioural phenotype. Maternal weight is shown in SD units, while behaviours are observed units except for Freezings (for which counts have been square root-transformed)

We find support for significant maternal effects mediated by *maternal weight*, *brood size* and/or their interaction on all juvenile behaviours (Fig. 1, Table 3). Juvenile offspring born to heavier mothers, on average, have a significantly shorter *traklength* and a non-significant trend towards lower *activity* (Table 3). Juveniles from larger broods covered more tank area. For *time in middle zone*, there was a significant interaction between brood size and maternal weight. Visualising the predictions from this model shows that while *maternal weight* has no effect on juvenile *time in middle zone* at an average brood size, the predicted relationship is negative for small *brood sizes* and weakly positive for large ones (Fig. 1).

**Table 3:** Estimated effects of brood size (BS, number of fish) and maternal weight (MW, g) and their interaction (BS:MW) on offspring behaviours at juvenile and adult stages

		Full model				Full model plus offspring standard length			
Trait	Fixed effect	Effect size	DF	*F*	*P*	Effect size	DF	*F*	*P*
**Juvenile**									
*Tracklength*	**BS**	**0.062 (0.052)**	**1, 188.7**	**0.92**	**0.338**	**0.231 (0.057)**	**1, 257.8**	**14.68**	<0.001*
	MW	−0.118 (0.052)	1, 57.3	4.79	0.033*	−0.161 (0.051)	1, 55.1	9.11	0.004*
	BS−MW	−0.032 (0.042)	1, 110.3	0.58	0.447	−0.050 (0.041)	1, 104.9	1.53	0.219
	OL	—	—	—	—	0.236 (0.039)	1, 603.7	37.70	<0.001*
*Activity*	**BS**	**0.035 (0.055)**	**1, 208.0**	**0.08**	**0.779**	**0.239 (0.060)**	**1, 279.3**	**13.86**	<0.001*
	**MW**	−**0.114 (0.057)**	**1, 57.9**	**3.63**	**0.062**	−**0.168 (0.055)**	**1, 55.6**	**8.31**	0.006*
	BS−MW	−0.042 (0.045)	1, 122.8	0.88	0.351	−0.066 (0.043)	1, 116.6	2.34	0.129
	OL	—	—	—	—	0.286 (0.039)	1, 612.1	54.75	<0.001*
*Area covered*	BS	0.198 (0.072)	1, 237.1	11.08	0.001*	0.204 (0.081)	1, 320.5	9.25	0.003*
	MW	0.020 (0.076)	1, 64.6	0.04	0.834	0.019 (0.077)	1, 65.0	0.03	0.855
	BS-MW	0.035 (0.058)	1, 141.4	0.369	0.545	0.035 (0.059)	1, 140.6	0.35	0.555
	OL	—	—	—	—	0.008 (0.051)	1, 616.6	0.03	0.869
*Time in middle*	**BS**	−**0.057 (0.064)**	**1, 141.8**	**0.01**	**0.917**	−**0.226 (0.073)**	**1, 199.7**	**5.56**	0.019*
	MW	−0.025 (0.059)	1, 51.7	0.54	0.466	0.015 (0.058)	1, 49.9	0.02	0.901
	BS-MW	0.103 (0.049)	1, 72.6	4.37	0.040*	0.119 (0.048)	1, 68.1	6.08	0.016*
	OL	—	—	—	—	−0.237 (0.053)	1, 564.2	20.22	<0.001*
*Freezings*	**BS**	−**0.075 (0.059)**	**1, 177.5**	**1.90**	**0.170**	−**0.156 (0.067)**	**1, 243.1**	**5.96**	0.015*
	MW	0.077 (0.058)	1, 55.6	1.76	0.190	0.096 (0.057)	1, 54.7	2.73	0.104
	BS-MW	0.001 (0.047)	1, 102.1	< 0.01	0.982	0.010 (0.046)	1, 95.7	0.05	0.831
	OL	—	—	—	—	−0.120 (0.046)	1, 596.0	6.89	0.009*
**Adult**									
*Tracklength*	**BS**	−**0.070 (0.050)**	**1, 217**	**4.31**	0.039*	−**0.008 (0.050)**	**1, 229.4**	**0.617**	**0.433**
	MW	0.057 (0.49)	1, 64.6	1.53	0.220	0.060 (0.049)	1, 65.9	1.707	0.196
	BS-MW	−0.042 (0.038)	1, 166	1.24	0.268	−0.048 (0.037)	1, 173.6	1.664	0.199
	OL	—	—	—	—	0.173 (0.026)	1, 1028.8	43.160	<0.001*
*Activity*	**BS**	−**0.055 (0.048)**	**1, 194.5**	**5.46**	0.021*	**0.004 (0.049)**	**1, 202.9**	**1.104**	**0.295**
	MW	0.023 (0.044)	1, 65.2	0.35	0.555	0.030 (0.044)	1, 65.6	0.559	0.457
	BS-MW	−0.079 (0.036)	1, 130.9	4.69	0.032*	−0.084 (0.036)	1, 135.9	5.489	0.021*
	OL	—	—	—	—	0.170 (0.028)	1, 992.4	36.500	<0.001*
*Area covered*	**BS**	−**0.091 (0.046)**	**1, 616.1**	**2.04**	**0.150**	−**0.127 (0.047)**	**1, 576.2**	**4.915**	0.027*
	MW	0.085 (0.041)	1, 454.0	4.23	0.040*	0.078 (0.040)	1, 413.9	3.633	0.057
	BS-MW	0.053 (0.034)	1, 576.6	2.48	0.116	0.055 (0.033)	1, 538.8	2.801	0.095
	OL	—	—	—	—	−0.108 (0.028)	1, 939.1	15.080	<0.001*
*Time in middle*	**BS**	−**0.038 (0.048)**	**1, 436.7**	**0.12**	**0.732**	−**0.131 (0.046)**	**1, 351.2**	**6.447**	0.012*
	MW	0.005 (0.042)	1, 300.0	0.02	0.897	−0.025 (0.039)	1, 222.6	0.414	0.520
	BS-MW	0.039 (0.036)	1, 425.5	1.23	0.269	0.043 (0.033)	1, 304.0	1.728	0.190
	OL	—	—	—	—	−0.253 (0.029)	1 1028.7	74.360	<0.001*
*Freezings*	BS	0.013 (0.046)	1, 563.6	1.66	0.198	−0.001 (0.046)	1, 476.6	0.660	0.417
	MW	0.045 (0.041)	1, 529.0	1.21	0.272	−0.029 (0.040)	1, 493.5	0.500	0.480
	BS-MW	0.065 (0.034)	1, 637.0	3.75	0.053	0.055 (0.034)	1, 603.2	2.719	0.100
	OL	—	—	—	—	−0.037 (0.029)	1, 892.8	1.610	0.205

All estimates come from full (i.e., *V*_A_ + *V*_M_) models as described in the main text and then refitted with offspring standard length (OL) included as an additional fixed covariate. Effects that are significant at *P* = <0.05 under either model formulation are denoted by *. Bold font is used to highlight fixed effects that are significant under one formulation but not the other

In adults, there was a significant positive effect of *maternal weight* on *area covered*, while *brood size* negatively predicted *tracklength* and *activity* (Table 3). Adult *activity* is subject to a significant interaction between *maternal weight* and *brood size* (with maternal weight positively predicting *activity* for small broods but negatively for the largest ones; Fig. 1). Overall, these maternal effects show a tendency of being stronger in juveniles compared to adults (i.e., tendency for smaller effect size estimates in adult traits; Table 3). Moreover, in a qualitative sense the maternal trait(s) that significantly influence each observed behaviour differs between juveniles and adults (Table 3). For completeness, estimates of all other fixed effects from the full models can be found in Supplemental Table 3.

### Offspring length mediates maternal effects on offspring behaviour

In additional models, length had a positive effect on *tracklength* and *activity* and a negative effect on *time in middle zone* and *freezings* in juveniles. Similarly, in adults, *tracklength* and *activity* were positively influenced while both *area covered* and *time in middle zone* were negatively influenced by offspring length (Table 3). However, while this suggests relationships between risk-taking behaviour and size and/or growth, for juvenile behaviours, the inclusion of length as a predictor did not notably reduce the estimated effects of *maternal weight* or *brood size* (in fact, effect size estimates increased in a number of cases; Table 3). For adult *tracklength* and *activity*, however, the addition of length to the model resulted in a large drop in the magnitude of *brood size* effect. This suggests that maternal brood size effects on behaviour of adult offspring may well be mediated by intermediate effects on size.

### Maternal genetic and grand-maternal effects

Meaningful testing for heritable variation and/or maternal identity effects for the *brood size* maternal trait was not possible due to insufficient numbers of broods from females with known parentage themselves. However, the animal model analysis of *maternal weight* indicated that both additive genetic and maternal identity effects are major components of variance in this trait (*h*^2^ = 0.62 (0.06), *χ*^2^_0,1_ = 107.26, *P* = <0.001; *m*^2^ = 0.30 (0.07), *χ*^2^_0,1_ = 74.36, *P* = <0.001), while the permanent environment effect was bound to zero.

## Discussion

Here we estimated maternal and additive genetic effects on offspring risk-taking behaviour in the guppy, and asked whether the importance of these two sources of among-individual variation changes over ontogeny. Below we discuss the ontogenetic patterns in maternal and additive genetic effects in more detail, before further considering the consequences of genetic variance in maternal weight. We place our results in the context of the wider quantitative genetics literature, and discuss their implications for understanding the evolutionary dynamics of personality in this species.

### Maternal and additive genetic effects both contribute to variation in risk-taking behaviour

We found that maternal effects for offspring risk-taking behaviour are present in this population of guppies. This was evidenced by estimates of the maternal identity variance component and by the estimated effects on offspring behaviour of maternal weight and brood size. Heritabilities were estimated at zero for juvenile behaviours and, for adult OFT traits, were low to moderate relative to those published in the personality literature (van Oers et al. 2005; Dingemanse et al. 2009; Niemelä et al. 2013; Petelle et al. 2015). We highlight that, for juvenile traits, heritability estimates made in the assumed absence of maternal identity effects were much higher than those from the full models since almost all among-family variance was partitioned as additive. For adult traits, *V*_M_ accounted for a smaller proportion of total phenotypic variance in the full models (discussed further below). Accordingly, *h*^2^ estimates were not increased as much by assuming an absence of maternal identity effects. More generally, these results demonstrate the point that failing to account for maternal effects in animal models can upwardly bias estimates of additive genetic variance (Falconer and Mackay 1996; Kruuk 2004; Wilson et al. 2010; Mcglothlin and Galloway 2013). To date, few studies of personality have explicitly tested for maternal effects (but see e.g., Taylor et al. 2012), and the possibility certainly exists that our emerging view of additive genetic contributions to behavioural variation is biased. However, as a partial caveat to our current results, we highlight again that brood size necessarily determines early rearing density (i.e., prior to tagging) in our experimental design. Although early life rearing density was found to have no impact on bold type behaviours in a recent study of the related fish *Xiphophorus birchmanni* (Boulton et al. 2018), the situation could be different here. Thus, brood size potentially integrates maternal influences across pre- and post-natal periods. We note that in natural populations, dispersal coupled to an absence of post-natal care likely limit the potential for post-natal maternal effects.

### Changing importance of maternal and additive genetic effects over ontogeny

Our results are consistent with the prediction made that maternal effects on offspring traits will decrease with (offspring) age. While acknowledging that separation of *V*_M_ and *V*_A_ can be problematic in some data structures, under the full model, *m*^2^ estimates for each trait were higher than for the corresponding adult behaviours (for which the *V*_M_ explained very little to no variance in all but *tracklength*). A pattern of declining maternal effects with age is also seen in the effects of maternal weight and brood size on offspring behaviour, which are consistently stronger in juveniles than adults. This matches the general pattern of age-related declines in maternal effects in the literature. For instance, Houde et al. (2013) found that maternal effects on survival declined during development from egg to fry stages in Atlantic salmon (*Salmo salar*). Similarly, maternal effects decline with age for body size in *P. parae* (a close relative of the Trinidadian guppy; Lindholm et al 2006) and the lemon shark (*Negaprion brevirostris*; Dibattista et al. 2009), while maternal identity explains more variation in pathogen resistance in younger than in older whitefish (*Coregonus palaea*) (Clark et al. 2014). It is generally held that this pattern arises because while the point of last maternal influence becomes more distant in time, other sources of trait variation continue to be experienced, and in some cases new influences on phenotype arise (e.g., changes in gene expression after sexual maturity).

Despite this general pattern, some maternal effects were detected on adult behaviours. Interestingly, there was little qualitative correspondence in the specific maternal traits that significantly influenced a given behaviour in juveniles versus adults. For example, maternal weight significantly affected juvenile but not adult *tracklength*, while *area covered* was affected by brood size in juveniles but maternal weight in adults. This suggests that not only does the overall maternal influence on offspring behaviour wane over ontogeny, but that age-specific maternal effects could arise through different pathways. In addition, both tracklength and activity had non-zero amounts of variance explained by maternal identity (significantly so in the former) compared to the other offspring traits with zero maternal identity effect. This difference suggests that the traits are not all equivalent proxies of a single underlying personality axis here. Indeed, in a previous study of independent data, we found that tracklength and activity capture among-individual variation that might be better interpreted as stress-responsiveness, while pattern of variation in the remaining are more aligned with expectations under a simple ‘boldness’ paradigm (White et al. 2016). Using the current adult data, multivariate modelling of both sexes combined, and of males and females separately corroborates this interpretation (White et al. submitted manuscript).

As well as declining maternal effects, we predicted that additive genetic contributions to behavioural variation would increase with age. This pattern is well documented for a range of trait types in the literature (Atchley and Zhu 1997; Houle 1998; Wilson and Réale 2005; Lindholm et al. 2006) and is also supported in our study. More specifically, our estimates of *h*^2^ clearly uphold this prediction and we note that robust statistical support for additive genetic variance is only present in adult behaviours. While not directly relevant to current hypotheses, our analysis also shows that a lot of among-individual variance described previously by us and others in these OFT traits is explained by neither additive nor maternal effects. The source of this behavioural variation is unknown, and we have controlled as much as possible for shared environment using common water supplies and identical tanks for each family/group. Nonetheless, among-individual variance can arise from uncontrolled (and unmodelled) aspects of the physical environment or potentially from the social environment (Lindholm et al. 2006; Moretz et al. 2007; Krause et al. 2010; Piyapong et al. 2010). In fact, the *Group* random effect is significant for all traits in adults, consistent with the latter being an important determinant of behaviour here.

### Offspring length as a mediator of maternal effects

Given known maternal effects on offspring size and growth in guppies (Reznick et al. 1996; Bashey 2006) and the widely reported size-dependence of personality (Brown and Braithwaite 2004; Rödel and Meyer 2011; Biro and Sampson 2015), offspring size provides a plausible link in the mechanistic pathway between maternal traits and offspring behaviours they influence. Somewhat consistent with this hypothesis, we did find that adding length as a fixed predictor led to large decreases in the estimated effect of brood size on tracklength and *activity* in adults. We also note that, in accordance with earlier studies (Reznick et al. 1996; Bashey 2006), offspring born into larger broods are on average smaller at birth and when measured as juveniles (results not shown). However, while length significantly predicted four of the five juvenile behaviours and all of the adult traits, its inclusion as a covariate did not, with the two exceptions noted above, result in a decrease to maternal effect estimates. This indicates that maternal effects on behaviour may be mediated through offspring growth in some cases, but that additional pathways (for instance, hormonal transfer—Rokka et al. 2014; Hinde et al. 2015, or stochastic developmental events (Bierbach et al. 2017)) are also involved.

### Maternal genetic and grand-maternal effects on risk-taking behaviour

As discussed above, our analyses indicate maternal weight and brood size to be significant sources of maternal effects on offspring behaviour. Furthermore, we found that maternal weight has a significant additive genetic component of variance, and is thus expected to generate maternal genetic effects (McAdam et al. 2013). In the presence of maternal genetic effects, offspring personality traits will respond not just to direct selection on them, but also to any selection on the maternal trait (in this case weight) in the previous generation (Kirkpatrick and Lande 1989). Covariance between additive and maternal genetic effects can also occur, potentially constraining phenotypic evolution and maintaining genetic (and therefore phenotypic) variation in both maternal and offspring traits (Kirkpatrick and Lande 1989; Wilson et al. 2005; Räsänen and Kruuk 2007). Thus, the presence of maternal genetic effects alters expectations for evolutionary change relative to those based on direct selection alone. Here our estimated heritabilities alone would suggest adult behaviours have greater potential for adaptive evolution that juvenile ones. However, this ignores the possible role of maternal genetic effects that can be large. For instance, McAdam and Boutin (2004) showed that failing to account for selection on litter size (the maternal trait) in the red squirrel (*Tamiasciurus hudsonicus*) led to a predicted change in offspring size that was five times lower than the observed rate.

In the present case, the relationship between risk-taking behaviour and fitness is unknown, so it is difficult to comment on the extent of direct selection on them in juveniles or adults in wild populations. However, selection on female (maternal) weight is expected. Like many fish species, female guppies exhibit indeterminate growth, with fecundity increasing as a function of size (Bronikowski et al. 2002) and, when given the choice, male guppies will choose to mate with larger females (Dosen and Montgomerie 2004; Herdman et al. 2004). Thus, we can at least speculate that the evolution of personality traits in guppies will depend on selection on size through maternal fitness, particularly at the juvenile stage where maternal influence is strongest, highlighting another mechanism by which morphological and behavioural traits may co-evolve.

Finally, not only is maternal weight heritable, but we found evidence that it is itself subject to maternal effects, manifest as a significant estimate of *V*_M_. Accepting that maternal weight does causally influence offspring behaviour, this actually implies the possibility of grand-maternal effects on personality (Mcglothlin and Galloway 2013). In *Drosophila*, both maternal and grand-maternal age influenced offspring viability and spider mite (*Tetranychus urticae*) offspring dispersal distance is affected by the density that both maternal and grand-maternal generations experienced (Hercus and Hoffmann 2000; Bitume et al. 2014). Very few studies outside of domestic animal breeding have looked into grand-maternal effects, however, owing to the difficulty in collecting multigenerational pedigree data and none to our knowledge have looked at personality in this regard.

### Summary

We found that both additive genetic and maternal effects are important determinants of risk-taking behaviour traits in guppies, although the former are only evident in adult fish. Not accounting for the maternal effects resulted in much higher *h*^2^ estimates in some cases raising the possibility that current estimates for personality traits are upwardly biased. Robust evidence of additive genetic variance was found for adult traits but maternal effects are also present, though with generally much smaller effect sizes than in juveniles. In contrast, our models did not provide statistical support for additive variance in juvenile behaviours. Rather our results indicate, among-family variance arises principally from maternal identity effects, as well as maternal effects occurring via variation in maternal weight and brood size. Moreover, the specific maternal traits influencing offspring behaviour differed between juveniles and adults, suggestive of a shift in the mechanism through which maternal effects influence behaviour over ontogeny. Offspring size is a plausible candidate trait for mediating maternal effects on behaviour in some cases but not all. Our study highlights the benefit of employing the hybrid approach for estimating maternal effects at different stages over offspring ontogeny, and of using animal models to estimate both the additive genetic structure and maternal effects for personality traits. We suggest that wider efforts to characterise maternal effects, and especially to test their genetic basis, could greatly benefit our understanding of the evolutionary dynamics of animal personality.

### Data archiving

The research data supporting this publication are openly available from the University of Exeter’s institutional repository at: https://doi.org/10.24378/exe.225.

## Electronic supplementary material

Supplemental table 1

Supplemental table 2

Supplemental table 3

Appendix 1

Appendix 2
