# Supplementary material for: Evolutionary genetics of personality in the Trinidadian guppy I: maternal and additive genetic effects across ontogeny
Source: Heredity (Edinb). 2018 May 17;122(1):1–14. doi: 10.1038/s41437-018-0082-1 (PMC6288082; doi:10.1038/s41437-018-0082-1)
Supplement: Supplementary file 1 — Supplemental table 1 [file 41437_2018_82_MOESM1_ESM.docx]

Supplemental table 1: Likelihood ratio tests comparison full models (as described in main text) that included both additive genetic and maternal identity effects fitted vs ‘null’ models with identical fixed effects but neither of these random effects to model among family variance.

| Trait | Juvenile | | Adult | |
| --- | --- | --- | --- | --- |
|  | χ^2^_2_ | P | χ^2^_2_ | P |
| *Tracklength* | 47.40 | <0.001 | 40.23 | <0.001 |
| *Activity* | 54.44 | <0.001 | 42.12 | <0.001 |
| *Area covered* | 69.90 | <0.001 | 21.42 | <0.001 |
| *Time in middle* | 13.82 | <0.001 | 13.56 | <0.001 |
| *Freezings* | 35.95 | <0.001 | 27.07 | <0.001 |
