# Supplementary material for: Evolutionary genetics of personality in the Trinidadian guppy I: maternal and additive genetic effects across ontogeny
Source: Heredity (Edinb). 2018 May 17;122(1):1–14. doi: 10.1038/s41437-018-0082-1 (PMC6288082; doi:10.1038/s41437-018-0082-1)
Supplement: Supplementary file 2 — Supplemental table 2 [file 41437_2018_82_MOESM2_ESM.docx]

Supplemental table 2: Estimated variance components and associated ratios to phenotypic variance in full models (containing random additive genetic and maternal identity effects) and in V_A_-only models (with no maternal identity effects). Standard errors are shown in parentheses except where a component is bound to zero (see main text for details).

Full model

| Trait | V_A_ | V_M_ | V_PE_ | V_Group_ | V_R_ | h^2^ | m^2^ | pe^2^ | Group^2^ |
| --- | --- | --- | --- | --- | --- | --- | --- | --- | --- |
| **Juvenile** |  |  |  |  |  |  |  |  |  |
| *Tracklength* | 0.000 | 0.096 (0.033) | - | - | 0.469 (0.028) | 0.000 | 0.170 (0.049) | - | - |
| *Activity* | 0.000 | 0.134 (0.043) | - | - | 0.474 (0.028) | 0.000 | 0.220 (0.057) | - | - |
| *Area covered* | 0.000 | 0.257 (0.077) | - | - | 0.756 (0.045) | 0.000 | 0.254 (0.059) | - | - |
| *Time in middle* | 0.000 | 0.098 (0.042) | - | - | 0.907 (0.053) | 0.000 | 0.097 (0.039) | - | - |
| *Freezings* | 0.000 | 0.113 (0.040) | - | - | 0.634 (0.037) | 0.000 | 0.151 (0.047) | - | - |
| **Adult** |  |  |  |  |  |  |  |  |  |
| *Tracklength* | 0.056 (0.045) | 0.079 (0.037) | 0.215 (0.034) | 0.043 (0.019) | 0.423 (0.014) | 0.068 (0.055) | 0.097 (0.042) | 0.263 (0.042) | 0.053 (0.023) |
| *Activity* | 0.164 (0.055) | 0.021 (0.023) | 0.182 (0.040) | 0.023 (0.014) | 0.504 (0.017) | 0.184 (0.058) | 0.023 (0.026) | 0.204 (0.046) | 0.026 (0.015) |
| *Area covered* | 0.167 (0.050) | 0.000 | 0.114 (0.037) | 0.155 (0.045) | 0.587 (0.020) | 0.163 (0.046) | 0.000 | 0.111 (0.038) | 0.151 (0.038) |
| *Time in middle* | 0.158 (0.056) | 0.000 | 0.237 (0.044) | 0.026 (0.015) | 0.534 (0.018) | 0.165 (0.055) | 0.000 | 0.248 (0.048) | 0.027 (0.016) |
| *Freezings* | 0.202 (0.054) | 0.000 | 0.093 (0.039) | 0.021 (0.013) | 0.662 (0.022) | 0.206 (0.051) | 0.000 | 0.096 (0.041) | 0.022 (0.013) |

V_A_-only model

| Trait | V_A_ | V_PE_ | V_Group_ | V_R_ | h^2^ | pe^2^ | Group^2^ |
| --- | --- | --- | --- | --- | --- | --- | --- |
| **Juvenile** |  |  |  |  |  |  |  |
| *Tracklength* | 0.252 (0.089) | - | - | 0.348 (0.055) | 0.420 (0.122) | - | - |
| *Activity* | 0.357 (0.120) | - | - | 0.300 (0.069) | 0.543 (0.138) | - | - |
| *Area covered* | 0.674 (0.208) | - | - | 0.422 (0.116) | 0.615 (0.136) | - | - |
| *Time in middle* | 0.174 (0.087) | - | - | 0.829 (0.074) | 0.173 (0.081) | - | - |
| *Freezings* | 0.278 (0.104) | - | - | 0.499 (0.068) | 0.358 (0.114) | - | - |
| **Adult** |  |  |  |  |  |  |  |
| *Tracklength* | 0.120 (0.037) | 0.186 (0.030) | 0.065 (0.024) | 0.424 (0.014) | 0.151 (0.045) | 0.234 (0.039) | 0.082 (0.028) |
| *Activity* | 0.178 (0.050) | 0.178 (0.038) | 0.025 (0.014) | 0.504 (0.017) | 0.201 (0.052) | 0.201 (0.044) | 0.028 (0.016) |
| *Area covered* | 0.167 (0.050) | 0.114 (0.037) | 0.155 (0.045) | 0.587 (0.020) | 0.163 (0.046) | 0.111 (0.038) | 0.151 (0.038 |
| *Time in middle* | 0.158 (0.056) | 0.237 (0.044) | 0.026 (0.015) | 0.534 (0.018) | 0.165 (0.055) | 0.248 (0.048) | 0.027 (0.016) |
| *Freezings* | 0.202 (0.054) | 0.093 (0.039) | 0.021 (0.013) | 0.662 (0.022) | 0.206 (0.051) | 0.096 (0.041) | 0.022 (0.013) |
