# Supplementary material for: Evolutionary genetics of personality in the Trinidadian guppy I: maternal and additive genetic effects across ontogeny
Source: Heredity (Edinb). 2018 May 17;122(1):1–14. doi: 10.1038/s41437-018-0082-1 (PMC6288082; doi:10.1038/s41437-018-0082-1)
Supplement: Supplementary file 3 — Supplemental table 3 [file 41437_2018_82_MOESM3_ESM.docx]

Supplemental table 3 – Fixed effect estimates with associated statistical inference for juvenile and adult behavioural traits. All estimates are from “Full models” as described in main text without inclusion of offspring standard length as a covariate

| Trait | Fixed effect | Effect size (SE) | DF | F | P |
| --- | --- | --- | --- | --- | --- |
| Juv Tracklength | Generation 1 | 0.000 | 1, 36.3 | 11.58 | 0.002 |
|  | Generation 2 | -0.404 ( 0.119) |  |  |  |
|  | Order 1 | 0.000 | 25, 587.0 | 1.26 | 0.179 |
|  | Order2 | 0.346 (0.128) |  |  |  |
|  | Order 3 | 0.374 (0.132) |  |  |  |
|  | Order 4 | 0.372 (0.134) |  |  |  |
|  | Order 5 | 0.362 (0.135) |  |  |  |
|  | Order 6 | 0.206 (0.138) |  |  |  |
|  | Order 7 | 0.417 (0.140) |  |  |  |
|  | Order 8 | 0.301 (0.144) |  |  |  |
|  | Order 9 | 0.548 (0.151) |  |  |  |
|  | Order 10 | 0.378 (0.158) |  |  |  |
|  | Order 11 | 0.404 (0.168) |  |  |  |
|  | Order 12 | 0.473 (0.168) |  |  |  |
|  | Order 13 | 0.305 (0.178) |  |  |  |
|  | Order 14 | 0.383 (0.191) |  |  |  |
|  | Order 15 | 0.137 (0.200) |  |  |  |
|  | Order 16 | 0.545 (0.218) |  |  |  |
|  | Order 17 | 0.349 (0.218) |  |  |  |
|  | Order 18 | -0.029 (0.226) |  |  |  |
|  | Order 19 | 0.503 (0.244) |  |  |  |
|  | Order 20 | 0.404 (0.255) |  |  |  |
|  | Order 21 | 0.210 (0.254) |  |  |  |
|  | Order 22 | 0.087 (0.302) |  |  |  |
|  | Order 23 | 0.424 (0.416) |  |  |  |
|  | Order 24 | 0.670 (0.416) |  |  |  |
|  | Order 25 | -0.350 (0.504) |  |  |  |
|  | Order 26 | 1.007 (0.707) |  |  |  |
|  | Age | -0.050 (0.042) | 1, 219.2 | 1.38 | 0.241 |
|  | Temp | 0.603 (0.054) | 1, 65.5 | 122.90 | <0.001 |
| Juv Activity | Generation 1 | 0.000 | 1, 35.1 | 5.53 | 0.024 |
|  | Generation 2 | -0.314 (0.134) |  |  |  |
|  | Order 1 | 0.000 | 25, 583.3 | 1.13 | 0.306 |
|  | Order 2 | 0.287 (0.129) |  |  |  |
|  | Order 3 | 0.347 (0.132) |  |  |  |
|  | Order 4 | 0.342 (0.135) |  |  |  |
|  | Order 5 | 0.310 (0.136) |  |  |  |
|  | Order 6 | 0.167 (0.140) |  |  |  |
|  | Order 7 | 0.426 (0.142) |  |  |  |
|  | Order 8 | 0.238 (0.145) |  |  |  |
|  | Order 9 | 0.556 (0.153) |  |  |  |
|  | Order 10 | 0.314 (0.159) |  |  |  |
|  | Order 11 | 0.345 (0.169) |  |  |  |
|  | Order 12 | 0.453 (0.169) |  |  |  |
|  | Order 13 | 0.283 (0.180) |  |  |  |
|  | Order 14 | 0.421 (0.193) |  |  |  |
|  | Order 15 | 0.163 (0.202) |  |  |  |
|  | Order 16 | 0.532 (0.220) |  |  |  |
|  | Order 17 | 0.401 (0.220) |  |  |  |
|  | Order 18 | 0.087 (0.228) |  |  |  |
|  | Order 19 | 0.482 (0.245) |  |  |  |
|  | Order 20 | 0.476 (0.257) |  |  |  |
|  | Order 21 | 0.301 (0.256) |  |  |  |
|  | Order 22 | 0.188 (0.304) |  |  |  |
|  | Order 23 | 0.479 (0.419) |  |  |  |
|  | Order 24 | 0.601 (0.419) |  |  |  |
|  | Order 25 | -0.236 (0.508) |  |  |  |
|  | Order 26 | 1.152 (0.712) |  |  |  |
|  | Age | 0.002 ( 0.044) | 1, 247.6 | <0.01 | 0.962 |
|  | Temp | 0.604 (0.060) | 1, 69.9 | 102.60 | <0.001 |
| Juv Area covered | Generation 1 | 0.000 | 1, 37.8 | 7.42 | 0.010 |
|  | Generation 2 | 0.494 (0.181) |  |  |  |
|  | Order 1 | 0.000 | 25, 584.7 | 1.40 | 0.097 |
|  | Order 2 | -0.123 (0.163) |  |  |  |
|  | Order 3 | 0.024 (0.167) |  |  |  |
|  | Order 4 | -0.145 (0.170) |  |  |  |
|  | Order 5 | -0.126 (0.173) |  |  |  |
|  | Order 6 | -0.217 (0.176) |  |  |  |
|  | Order 7 | -0.351 (0.179) |  |  |  |
|  | Order 8 | -0.529 (0.183) |  |  |  |
|  | Order 9 | -0.103 (0.193) |  |  |  |
|  | Order 10 | -0.395 (0.202) |  |  |  |
|  | Order 11 | -0.417 (0.214) |  |  |  |
|  | Order 12 | -0.287 (0.214) |  |  |  |
|  | Order 13 | 0.154 (0.227) |  |  |  |
|  | Order 14 | -0.115 (0.244) |  |  |  |
|  | Order 15 | -0.382 (0.255) |  |  |  |
|  | Order 16 | 0.196 (0.278) |  |  |  |
|  | Order 17 | -0.433 (0.279) |  |  |  |
|  | Order 18 | -0.664 (0.288) |  |  |  |
|  | Order 19 | -0.197 (0.310) |  |  |  |
|  | Order 20 | -0.389 (0.325) |  |  |  |
|  | Order 21 | -0.375 (0.324) |  |  |  |
|  | Order 22 | -0.528 (0.385) |  |  |  |
|  | Order 23 | -0.296 (0.530) |  |  |  |
|  | Order 24 | 0.078 (0.530) |  |  |  |
|  | Order 25 | -1.507 (0.641) |  |  |  |
|  | Order 26 | -1.244 (0.900) |  |  |  |
|  | Age | 0.129 (0.057) | 1, 282.8 | 5.14 | 0.024 |
|  | Temp | -0.030 (0.079) | 1, 80.4 | 0.14 | 0.705 |
| Juv Time in middle | Generation 1 | 0.000 | 1, 32.8 | <0.01 | 0.985 |
|  | Generation 2 | 0.002 (0.127) |  |  |  |
|  | Order 1 | 0.000 | 25, 591.3 | 1.01 | 0.457 |
|  | Order 2 | -0.171 (0.179) |  |  |  |
|  | Order 3 | -0.214 (0.183) |  |  |  |
|  | Order 4 | -0.227 (0.185) |  |  |  |
|  | Order 5 | -0.400 (0.188) |  |  |  |
|  | Order 6 | -0.183 (0.192) |  |  |  |
|  | Order 7 | -0.371 (0.194) |  |  |  |
|  | Order 8 | -0.448 (0.199) |  |  |  |
|  | Order 9 | -0.420 (0.210) |  |  |  |
|  | Order 10 | -0.211 (0.219) |  |  |  |
|  | Order 11 | -0.642 (0.233) |  |  |  |
|  | Order 12 | -0.579 (0.232) |  |  |  |
|  | Order 13 | -0.030 (0.247) |  |  |  |
|  | Order 14 | -0.189 (0.265) |  |  |  |
|  | Order 15 | -0.231 (0.278) |  |  |  |
|  | Order 16 | -0.121 (0.302) |  |  |  |
|  | Order 17 | -0.444 (0.303) |  |  |  |
|  | Order 18 | -0.119 (0.313) |  |  |  |
|  | Order 19 | -0.452 (0.338) |  |  |  |
|  | Order 20 | -0.170 (0.354) |  |  |  |
|  | Order 21 | -0.176 (0.353) |  |  |  |
|  | Order 22 | -0.717 (0.420) |  |  |  |
|  | Order 23 | -0.375 (0.578) |  |  |  |
|  | Order 24 | -0.462 (0.578) |  |  |  |
|  | Order 25 | -1.027 (0.700) |  |  |  |
|  | Order 26 | -2.327 (0.981) |  |  |  |
|  | Age | 0.001 (0.052) | 1, 149.5 | <0.01 | 0.980 |
|  | Temp | -0.157 (0.061) | 1, 51.3 | 6.57 | 0.013 |
| Juv Freezings | Generation 1 | 0.000 | 1, 35.6 | 6.49 | 0.426 |
|  | Generation 2 | 0.106 (0.13) |  |  |  |
|  | Order 1 | 0.000 | 25, 587.5 | 0.91 | 0.591 |
|  | Order2 | -0.101 (0.149) |  |  |  |
|  | Order 3 | -0.197 (0.153) |  |  |  |
|  | Order 4 | -0.262 (0.155) |  |  |  |
|  | Order 5 | -0.242 (0.157) |  |  |  |
|  | Order 6 | -0.057 (0.161) |  |  |  |
|  | Order 7 | -0.205 (0.163) |  |  |  |
|  | Order 8 | -0.134 (0.167) |  |  |  |
|  | Order 9 | -0.310 (0.176) |  |  |  |
|  | Order 10 | -0.223 (0.183) |  |  |  |
|  | Order 11 | -0.390 (0.195) |  |  |  |
|  | Order 12 | -0.468 (0.195) |  |  |  |
|  | Order 13 | -0.207 (0.207) |  |  |  |
|  | Order 14 | -0.430 (0.222) |  |  |  |
|  | Order 15 | -0.359 (0.233) |  |  |  |
|  | Order 16 | -0.461 (0.253) |  |  |  |
|  | Order 17 | -0.614 (0.254) |  |  |  |
|  | Order 18 | -0.085 (0.262) |  |  |  |
|  | Order 19 | -0.473 (0.283) |  |  |  |
|  | Order 20 | -0.255 (0.296) |  |  |  |
|  | Order 21 | -0.045 (0.295) |  |  |  |
|  | Order 22 | -0.178 (0.351) |  |  |  |
|  | Order 23 | -0.643 (0.484) |  |  |  |
|  | Order 24 | -0.549 (0.484) |  |  |  |
|  | Order 25 | -0.203 (0.586) |  |  |  |
|  | Order 26 | -1.924 (0.821) |  |  |  |
|  | Age | -0.038 (0.048) | 1, 203.6 | 0.61 | 0.429 |
|  | Temp | -0.519 (0.061) | 1, 62.0 | 72.60 | <0.001 |
| Adult Tracklength | Generation 0 | 0.000 | 2, 132.1 | 5.336 | 0.006 |
|  | Generation 1 | 0.404 (0.138) |  |  |  |
|  | Generation 2 | 0.085 (0.155) |  |  |  |
|  | Order 1 | 0.259 (0.118) | 17, 2343.4 | 3.017 | <0.001 |
|  | Order2 | 0.404 (0.119) |  |  |  |
|  | Order 3 | 0.523 (0.135) |  |  |  |
|  | Order 4 | 0.509 (0.135) |  |  |  |
|  | Order 5 | 0.523 (0.136) |  |  |  |
|  | Order 6 | 0.504 (0.135) |  |  |  |
|  | Order 7 | 0.402 (0.136) |  |  |  |
|  | Order 8 | 0.429 (0.136) |  |  |  |
|  | Order 9 | 0.446 (0.137) |  |  |  |
|  | Order 10 | 0.498 (0.138) |  |  |  |
|  | Order 11 | 0.487 (0.139) |  |  |  |
|  | Order 12 | 0.405 (0.138) |  |  |  |
|  | Order 13 | 0.262 (0.140) |  |  |  |
|  | Order 14 | 0.332 (0.141) |  |  |  |
|  | Order 15 | 0.346 (0.147) |  |  |  |
|  | Order 16 | 0.049 (0.152) |  |  |  |
|  | Order 17 | 0.290 (0.784) |  |  |  |
|  | Repeat 0 | 0.000 | 4, 1704.1 | 12.340 | <0.001 |
|  | Repeat 1 | 0.598 (0.213) |  |  |  |
|  | Repeat 2 | 0.729 (0.215) |  |  |  |
|  | Repeat 3 | 0.8432 (0.219) |  |  |  |
|  | Repeat 4 | 0.796 (0.220) |  |  |  |
|  | Age | 0.046 (0.049) | 1, 132.9 | 0.866 | 0.354 |
|  | Temp | 0.110 (0.029) | 1, 1273.0 | 14.480 | <0.001 |
| Adult Activity | Generation 0 | 0.000 | 2, 111.4 | 2.083 | 0.129 |
|  | Generation 1 | 0.0803 (0.143) |  |  |  |
|  | Generation 2 | -0.155 (0.158) |  |  |  |
|  | Order 1 | 0.393 (0.131) | 17, 2366.4 | 3.3200 | <0.001 |
|  | Order2 | 0.559 (0.132) |  |  |  |
|  | Order 3 | 0.664 (0.149) |  |  |  |
|  | Order 4 | 0.706 (0.149) |  |  |  |
|  | Order 5 | 0.686 (0.149) |  |  |  |
|  | Order 6 | 0.682 (0.149) |  |  |  |
|  | Order 7 | 0.621 (0.150) |  |  |  |
|  | Order 8 | 0.615 (0.150) |  |  |  |
|  | Order 9 | 0.679 (0.151) |  |  |  |
|  | Order 10 | 0.732 (0.152) |  |  |  |
|  | Order 11 | 0.731 (0.153) |  |  |  |
|  | Order 12 | 0.630 (0.152) |  |  |  |
|  | Order 13 | 0.468 (0.154) |  |  |  |
|  | Order 14 | 0.586 (0.155) |  |  |  |
|  | Order 15 | 0.588 (0.161) |  |  |  |
|  | Order 16 | 0.242 (0.167) |  |  |  |
|  | Order 17 | -0.548 (0.846) |  |  |  |
|  | Repeat 0 | 0.000 | 4, 1696.9 | 10.890 | <0.001 |
|  | Repeat 1 | 0.535 (0.22) |  |  |  |
|  | Repeat 2 | 0.683 (0.225) |  |  |  |
|  | Repeat 3 | 0.776 (0.228) |  |  |  |
|  | Repeat 4 | 0.739 (0.230) |  |  |  |
|  | Age | 0.021 (0.046) | 1, 112.6 | 0.210 | 0.648 |
|  | Temp | 0.116 (0.030) | 1, 888.7 | 14.560 | <0.001 |
| Adult Area covered | Generation 0 | 0.000 | 2, 103.2 | 8.124 | <0.001 |
|  | Generation 1 | 0.061 (0.157) |  |  |  |
|  | Generation 2 | 0.640 (0.180) |  |  |  |
|  | Order 1 | 0.077 (0.129) | 17, 2423.4 | 0.6431 | 0.860 |
|  | Order2 | 0.157 (0.130) |  |  |  |
|  | Order 3 | 0.0865 (0.150) |  |  |  |
|  | Order 4 | 0.061 (0.150) |  |  |  |
|  | Order 5 | 0.109 (0.150) |  |  |  |
|  | Order 6 | 0.082 (0.150) |  |  |  |
|  | Order 7 | 0.032 (0.150) |  |  |  |
|  | Order 8 | 0.025 (0.151) |  |  |  |
|  | Order 9 | 0.018 (0.152) |  |  |  |
|  | Order 10 | 0.027 (0.153) |  |  |  |
|  | Order 11 | 0.103 (0.154) |  |  |  |
|  | Order 12 | 0.031 (0.154) |  |  |  |
|  | Order 13 | 0.044 (0.155) |  |  |  |
|  | Order 14 | 0.063 (0.157) |  |  |  |
|  | Order 15 | 0.041 (0.163) |  |  |  |
|  | Order 16 | 0.048 (0.169) |  |  |  |
|  | Order 17 | 0.886 (0.886) |  |  |  |
|  | Repeat 0 | 0.000 | 4, 1750.8 | 0.833 | 0.504 |
|  | Repeat 1 | 0.023 (0.254) |  |  |  |
|  | Repeat 2 | 0.0811 (0.256) |  |  |  |
|  | Repeat 3 | 0.085 (0.260) |  |  |  |
|  | Repeat 4 | 0.095 (0.262) |  |  |  |
|  | Age | 0.098 (0.059) | 1, 172.0 | 2.809 | 0.096 |
|  | Temp | 0.002 (0.034) | 1, 1538.3 | 0.003 | 0.954 |
| Adult Time in middle | Generation 0 | 0.000 | 2, 155.9 | 16.800 | <0.001 |
|  | Generation 1 | 0.483 (0.146) |  |  |  |
|  | Generation 2 | 0.906 (0.161) |  |  |  |
|  | Order 1 | -0.043 (0.137) | 17, 2365.0 | 1.741 | 0.030 |
|  | Order2 | -0.205 (0.138) |  |  |  |
|  | Order 3 | -0.222 (0.156) |  |  |  |
|  | Order 4 | -0.412 (0.156) |  |  |  |
|  | Order 5 | -0.295 (0.156) |  |  |  |
|  | Order 6 | -0.363 (0.156) |  |  |  |
|  | Order 7 | -0.291 (0.157) |  |  |  |
|  | Order 8 | -0.255 (0.157) |  |  |  |
|  | Order 9 | -0.369 (0.158) |  |  |  |
|  | Order 10 | -0.381 (0.159) |  |  |  |
|  | Order 11 | -0.338 (0.160) |  |  |  |
|  | Order 12 | -0.308 (0.160) |  |  |  |
|  | Order 13 | -0.230 (0.161) |  |  |  |
|  | Order 14 | -0.251 (0.162) |  |  |  |
|  | Order 15 | -0.333 (0.168) |  |  |  |
|  | Order 16 | -0.080 (0.174) |  |  |  |
|  | Order 17 | 1.154 (0.878) |  |  |  |
|  | Repeat 0 | 0.000 | 4, 1710.0 | 5.326 | <0.001 |
|  | Repeat 1 | -0.045 (0.229) |  |  |  |
|  | Repeat 2 | -0.201 (0.230) |  |  |  |
|  | Repeat 3 | -0.192 (0.234) |  |  |  |
|  | Repeat 4 | -0.127 (0.235) |  |  |  |
|  | Age | -0.145 (0.047) | 1, 115.4 | 9.55 | 0.003 |
|  | Temp | -0.006 (0.031) | 1, 853.3 | 0.043 | 0.835 |
| Adult Freezings | Generation 0 | 0.000 | 2, 192.7 | 4.137 | 0.017 |
|  | Generation 1 | 0.345 (0.144) |  |  |  |
|  | Generation 2 | 0.453 (0.158) |  |  |  |
|  | Order 1 | -0.471 (0.134) | 17, 2443.2 | 3.102 | <0.001 |
|  | Order2 | -0.699 (0.136) |  |  |  |
|  | Order 3 | -0.749 (0.157) |  |  |  |
|  | Order 4 | -0.833 (0.157) |  |  |  |
|  | Order 5 | -0.759 (0.157) |  |  |  |
|  | Order 6 | -0.747 (0.157) |  |  |  |
|  | Order 7 | -0.805 (0.157) |  |  |  |
|  | Order 8 | -0.766 (0.158) |  |  |  |
|  | Order 9 | -0.776 (0.159) |  |  |  |
|  | Order 10 | -0.813 (0.160) |  |  |  |
|  | Order 11 | -0.899 (0.161) |  |  |  |
|  | Order 12 | -0.882 (0.161) |  |  |  |
|  | Order 13 | -0.639 (0.162) |  |  |  |
|  | Order 14 | -0.809 (0.164) |  |  |  |
|  | Order 15 | -0.953 (0.171) |  |  |  |
|  | Order 16 | -0.569 (0.177) |  |  |  |
|  | Order 17 | -0.510 (0.927) |  |  |  |
|  | Repeat 0 | 0.000 | 4, 1742.2 | 9.857 | <0.001 |
|  | Repeat 1 | -0.040 (0.247) |  |  |  |
|  | Repeat 2 | -0.253 (0.248) |  |  |  |
|  | Repeat 3 | -0.253 (0.252) |  |  |  |
|  | Repeat 4 | -0.297 (0.253) |  |  |  |
|  | Age | -0.004 (0.044) | 1, 111.8 | 0.009 | 0.923 |
|  | Temp | -0.017 (0.033) | 1, 636.4 | 0.264 | 0.607 |
