# Supplementary material for: Evolutionary genetics of personality in the Trinidadian guppy I: maternal and additive genetic effects across ontogeny
Source: Heredity (Edinb). 2018 May 17;122(1):1–14. doi: 10.1038/s41437-018-0082-1 (PMC6288082; doi:10.1038/s41437-018-0082-1)
Supplement: Supplementary file 4 — Appendix 1 [file 41437_2018_82_MOESM4_ESM.docx]

*Appendix 1 Breeding design and pedigree management*

**Breeding design**

To create a pedigreed sub-population, female fish were haphazardly sampled from stock and isolated from male contact for 3 months. This was to minimise the chance of them carrying viable sperm from previous matings (see below). Following the 3-month isolation, females, along with males haphazardly taken from stock were tagged under anaesthetic (buffered MS222 solution) using visible implant elastomer (VIE) to allow individual identification. They were then assigned to breeding groups of 4 females to one male, housed in 15L breeding tanks (18.5cm x 37cm x 22cm). Females were inspected daily, and heavily gravid individuals (as determined from swollen abdomens and an enlarged ‘gravid spot’) were isolated in 2.8L brood tanks to give birth. Once a brood was produced, maternal standard length (measured from tip of snout to caudal peduncle, mm), weight and brood size were recorded. The female was then returned to the breeding tank (with offspring raised initially in the brood tank; see below). Any females that did not produce a brood within two weeks of being isolated were returned to their breeding tank. Any offspring born in the breeding tank were excluded from the experiment as we could not be sure of maternal identity.

The first generation of offspring produced (G1) comprised 566 individuals from 72 broods in total. These broods were produced by 54 female and 33 male individuals out of an initial 171(133 female and 38 male) sampled haphazardly from stock to represent out parental (P) generation. The G1 generation was produced in two breeding bouts, the first between April and November 2013 and the second between February and April 2014. A further offspring generation (G2) was then produced between February and July 2015, primarily using crosses between G1 fish (haphazardly sampled but ensuring no known inbreeding). Note that female G1 fish used in this way were isolated for 3 months as above. To increase the number of families we also crossed some G1 males to addition stock (P) females (again following isolation). Thus for some G2 it is the case that paternal but not maternal grandparents are known (see Appendix 2 figure). For G2 production we also altered the housing regime slightly as each female was kept in its own 2.8L tank, with a single male moved between 3 females in the breeding group on a weekly basis. This meant it was unnecessary to isolate females to collect broods, and removed the problem of unknown maternity for broods being produced in the larger tanks. A total of 25 females and 12 males contributed 281 G2 offspring from 34 broods.

Offspring were kept initially in their brood tanks before, at an average of 56 days, being moved as families to larger “grow on” tanks (15L, 18.5cm x 37cm x 22cm). Standard length was measured on each fish on the day of birth and at ages 7, 14, 28, 42, 56, 70 and 84 days, using Vernier callipers. Note, however, that individuals cannot be identified at juvenile stage, precluding individual level analyses of repeated measures data. At an average age of 132 days (range 59-226) all G1 and G2 fish were taken from their brood groups, individually tagged using visible implant elastomer (VIE) and placed into mixed-family groups of 16 mature adults (8 males and 8 females). Tagged groups were housed in 15L tanks (with dimensions as as described above). Note, that because individuals were not tagged until adulthood we cannot link the identity of those G1 fish that became parents of G2 fish to their juvenile phenotypic records. However, the family of these fish is known, so for each we added their identity code (as a tagged G1 parent) to the set of dummy codes (for untagged individuals) corresponding to that family. This allowed us to maintain the integrity of known pedigree links between G1 and G2 generations in our animal model analyses.

Thus, in total, we collected behavioural data (as described in main text) on 847 juvenile fish (G1 and G2 generations only) contained within a pedigree structure having a maximum depth of 3 generations, and 45 sire and 79 dam individuals. Behavioural data were collected on 841 adult fish, comprising P generation individuals (including those that did not contribute to the G1), as well as all G1 and G2 individuals that survived to maturity.

**Husbandry rationale and mitigation of pedigree error risk**

Female guppies can store viable sperm from previous matings for prolonged periods (up to several months). As such we acknowledge that our breeding strategy, in which females used were (almost certainly) non-virgin comes with some risk of introducing pedigree error (i.e. some paternity could come from males other than the assigned mating partner). To minimise this risk, females were isolated from males for a minimum of 3 months before use in crosses. After that time there was no offspring production and no females appearing gravid. As the gestation period for guppies is approximately 1 month, any brood produced by a female less than month after exposure to the designated male mating was discarded as an extra precaution to ensure pedigree accuracy.

Our rationale for taking this strategy here (and elsewhere, e.g., Boulton et al. 2016) was threefold. First, relative to the alternative of raising female virgins, isolating older stock females gave us faster access to; large numbers of females already held as stock; access to older, and thus larger, females expected to produce larger broods sand thus greater sample size; and, allowed us to build the multigenerational pedigree by utilising G1 females in the production of G2. Second, although sperm storage is well documented in guppies, our knowledge of the biology indicates this is unlikely to be a major source of paternity error in our experiment. Specifically, strong sperm precedence effects have been documents, even when matings are separated by an hour (rather than ≥ 3 months as here; Evan & Magurran, 2011), while storage also impairs sperm velocity (Gasparini *et al*. 2014), and, as a consequence, competitiveness (Boschetto, *et al.* 2011). Third, previous simulation studies (REFS) indicate that bias in quantitative genetic parameters caused by low levels of paternity will generally be low (e.g., Morrissey et al 2007; Morrissey and Wilson 2010). We note in additional that the same pedigree structure is used for both juveniles and adults here, so it is also difficult to envisage how any bias in parameter estimates that does occur could compromise the main comparisons being made.

Thus, while we stress that our quantitative genetic analyses make the standard assumption that the pedigree structure is known without error, we have taken multiple husbandry steps to ensure this assumption is reasonable and note that key comparisons and conclusions are expected to be robust to minor violations.

**References**

K. Boulton, G.G. Rosenthal, A.J. Grimmer, C.A. Walling, **A.J. Wilson** (2016). Sex-specific plasticity and genotype x sex interactions for age and size of maturity in the Sheepshead swordtail, Xiphophorus birchmanni. Journal of Evolutionary Biology, **29***, 645-656.*

Boschetto C, Gasparini C, Pilastro A. 2011 Sperm number and velocity affect sperm competition success in the guppy (Poecilia reticulata). *Behav. Ecol. Sociobiol*. **65**, 813–821.

Evans, J.P., Magurran, A. E., 2001. Patterns of sperm precedence and predictors of paternity in the Trinidadian guppy. *Proc. R. Soc.* **268**:719-724.

Gasparini C, Kelley JL, Evans JP. 2014 Male sperm storage compromises sperm motility in guppies. *Biol. Lett.* **10**: 20140681.

M.B. Morrissey, **A.J. Wilson**, J.M. Pemberton and M.M. Ferguson (2007). A framework for power and sensitivity analyses for studies of the quantitative genetics of natural populations, and a case study in Soay sheep (Ovis aries). Journal of Evolutionary Biology, **20**, 2309-2321.

M.B. Morrissey & **A.J. Wilson** (2010). pedantics, an R package for pedigree-based genetic simulation and pedigree manipulation, characterization and viewing. Molecular Ecology Resources, **10**, 711-719
