# Supplementary material for: Evolutionary genetics of personality in the Trinidadian guppy I: maternal and additive genetic effects across ontogeny
Source: Heredity (Edinb). 2018 May 17;122(1):1–14. doi: 10.1038/s41437-018-0082-1 (PMC6288082; doi:10.1038/s41437-018-0082-1)

Appendix 2: Visualisation of the three generation (parental, G1 & G2) guppy pedigree structure. Black dots represent individuals, blue lines denote sire-offspring links and red lines denote dam-offspring links. Note that to G2 fish were produced by crosses between unrelated G1 fish where possible, in some cases they were between G1 males and previously unused stock (ie parental) females of unknown parentage.


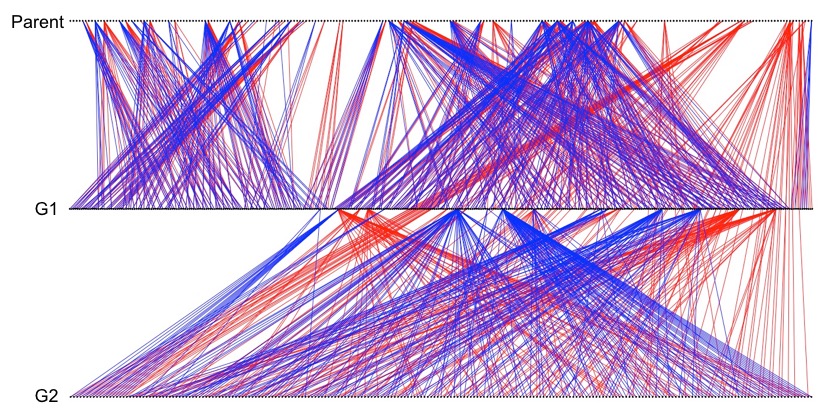

Supplement: Supplementary file 5 — Appendix 2 [file 41437_2018_82_MOESM5_ESM.docx]
